# Cooks That Kill

**Published:** 1875-06

**Authors:** 


### COOKS THAT KILL.

Somebody has said that "women are natural cooks." If this is true of women as a class, it is, at least, sadly untrue of women upon whom the average New-Yorker has to depend for the food upon which he strives to exist. These are botches, failures. We have been compelled, for many months, by the exigences of life, to eat beef-steak which the old Irish cook of a pretencious hotel here, had covered with warm water after the broiling process was ended! This was her regular system, and no expostulation would induce a change. The object was, as she explained, to make a lot of "nice water-gravy!" Of course her steak, with the most of the savory and valuable juices washed out of it, was about as palatable and nourishing as a damp rag. The meat was bought for the best and paid for at the highest rates, but it was ruined—utterly and absolutely—by the dire stupidity of an ignorant creature, who assumed to be a "cook" because she knew enough to expose food to the influences of heat, and to remove it from those influences before it was entirely burned up.

The fault, in this case, was with the person in whose charge this cooking automaton was supposed to be. The mistress was ignorant, and so the servant saw that her own ignorance had just as high a commercial value as skill. She was punctual; she never failed to have her ruined meat, her under-done vegetables, and her soggy, sour bread, on hand in time. So she held her place for years and made several thousand persons dyspeptics for life.

Why are we such stupid people? Why do we submit to the grossest of impositions—the imposition of misery and disease and wastefulness—on the part of those whom we pay to prepare our food.

When a man undertakes to build for himself a house, it is the general rule that he exercises the closest care that every portion of the structure shall be, in design and material, the best. He employs a capable architect, a thorough builder, selects stone, brick, mortar, and other components of his fabric, with a rigid scrutiny which leaves no doubt in his mind but that his dwelling will be a strong and lasting shelter. Then he decorates, furnishes, searches for ingenious devices of household convenience, and finally enters his new habitation secure in his belief of its excellence. Is it not strange that all his labor is done for a roof which may cover its owner only until to-morrow; for a home which the vicissitudes of fortune may wrest from him in a day, or which of his own choice he may abandon before the mortar is perfectly dry; while to the structure in which Providence has ordained he shall exist for a lifetime,—his body—but secondary consideration is given?

The trouble lies deep. We have no educated cooks. Our daughters are not taught how to cook accurately, as the chemist in the laboratory teaches his pupils. Our fashionable American society deems it altogether vulgar, if not quite dishonorable, to engage in household avocations. Thousands of families in New York, to-day, are wasting good food by bad cooking, while intelligent girls are barely keeping the breath of life in them, in shops and factories. Nothing pays better than cooking, and scarcely any employment is more fascinating to the mind of intelligence; yet the beautiful work, so attractive in its scientific aspects, so noble in its nature, and so satisfactory in its results, is left to stupid dolts who do not know the difference between albumen and gelatine, who cannot tell you what effect salt has on meat; who are, in short, utterly ignorant of the very first principles of the beautiful art of which they dishonestly assume to be competent exponents and professors.

